# *PHF7*, a novel male gene influences female fecundity and population growth in *Nilaparvata lugens* Stål (Hemiptera: Delphacidae)

**DOI:** 10.1038/s41598-017-11524-2

**Published:** 2017-09-14

**Authors:** Lin-Quan Ge, Ting Xia, Bo Huang, Hao-Tian Gu, Qi-Sheng Song, Guo-Qing Yang, Fang Liu, Jin-Cai Wu

**Affiliations:** 1 School of Plant Protection Yangzhou University, Yangzhou, 225009 P.R. China; 2 Division of Plant Sciences, University of Missouri, 1-31 Agriculture Building, Columbia, MO 65211 USA

## Abstract

PHF7 exhibits male-specific expression in early germ cells, germline stem cells and spermatogonia in insects, and its expression promotes spermatogenesis in germ cells when they are present in a male soma. However, the influence of male-specific PHF7 on female reproductive biology via mating remains unclear. Thus, we investigated the potential impacts of male PHF7, existed in seminal fluid of *Nilaparvata lugens* (*Nl*PHF7), on fecundity and population growth via mating. Our results revealed that suppressing male *NlPHF7* expression by RNAi led to decreases in body weight, soluble accessory gland protein content, arginine content, and reproductive organ development in males, resulting in significant reduction of oviposition periods and fecundity in females, and significant decrease in body weight, fat body and ovarian protein content, yeast-like symbionts abundance, ovarian development and vitellogenin gene expression in their female mating partners. Similarly, suppression of *NlPHF7* expression in males mated with the control female reduced population growth and egg hatching rate, but did not influence gender ratio. We infer that *Nl*PHF7 play a role important in stimulating female fecundity via mating. This study provides valuable information by identifying a potentially effective target gene for managing BPH population through RNAi.

## Introduction

In insects, male accessory gland (MAG) proteins are essential components of seminal fluids that act in influencing post-mating changes in female behavior, such as reduced sexual receptivity^1^, increased oviposition^2^ and increased sexual refractory periods^3^. MAG proteins act in sperm transport and storage, and because they also include antimicrobial peptides, they provide prophylactic protection for the female reproductive tract, activation and nourishment of sperms until fertilization^4^. Insect mating systems are evolved and tremendously varied traits. For a single example, MAG proteins do not induce refractoriness in all species^5^. MAG proteins also influence post-mating female physiology, stimulating oogenesis, ovulation and oviposition. The idea that males can influence their sexual partners via constituents of their seminal fluids is a strong insight into understanding the mating systems of many animal species and may have practical relevance for insect pest management^6^, especially for agricultural migratory insect pests.

The brown planthopper (BPH), *Nilaparvata lugens* (Stål) is a serious pest of rice crops in Asia and Australia^7^ and a classic example of an insecticide-induced resurgent pest^8^. Previous studies have demonstrated that insecticide treatments enhanced BPH MAG protein content^9^. In comparison with untreated males, females mated with insecticide-treated males led to increased fecundity^9^. The insecticides exerted effects on BPH reproduction appear to operate through a PHD finger protein 7 (*NlPHF7*) gene because it was up-regulated after triazophos (tzp)-treatment^10^. Unfortunately, the underlying relationship between insecticide-induced *NlPHF7* up-regulation and female reproduction has not been well elucidated. Nonetheless, there is a growing interest in exploring it as a BPH gene for its broad importance in animals and *Drosophila melanogaster*. PHF7, also known as testis development NYD-SP6, was discovered in research designed to identify new testicular development or spermatogenesis^11^. This work with a human testicular cDNA library yielded a gene encoding a protein-named NYD-SP6, which expresses in a wide range of tissues, particularly in the testis with a high expression level. It may play an important role in stimulating transcription involved in testicular development and/or spermatogenesis^11^. PHF7 is an important factor for male germline sexual identification in *Drosophila*, and *PHF7* exhibits male-specific expression in early germ cells, germline stem cells and spermatogonia^12^. The PHD finger protein has been found in many regulatory proteins from plants to animals, which is frequently associated with chromatin to mediate transcriptional regulation^13^. In a similar vein, BPH treated with the organophosphate tzp increased the expression of several proteins, including a spermatogenesis-associated protein like 5 (SPATA5) and testis development NYD-SP6 (PHF7)^4^. Suppression of *NlSPATA5* expression influenced male vitellogenin gene (*Nlvg*) expression and the number of eggs-laid by adult females via mating^14^.

Here we designed experiments to test our hypothesis that a single male gene product, *Nl*PHF7, influences parameters of female reproductive biology via mating. If this idea is supported by experimental results, the gene encoding *Nl*PHF7 could potentially serve as a target for RNAi based control of BPH in transgenic plants.

## Result

### Dietary ds*Nl*PHF7 reduces gene expression

**Figure 1:**
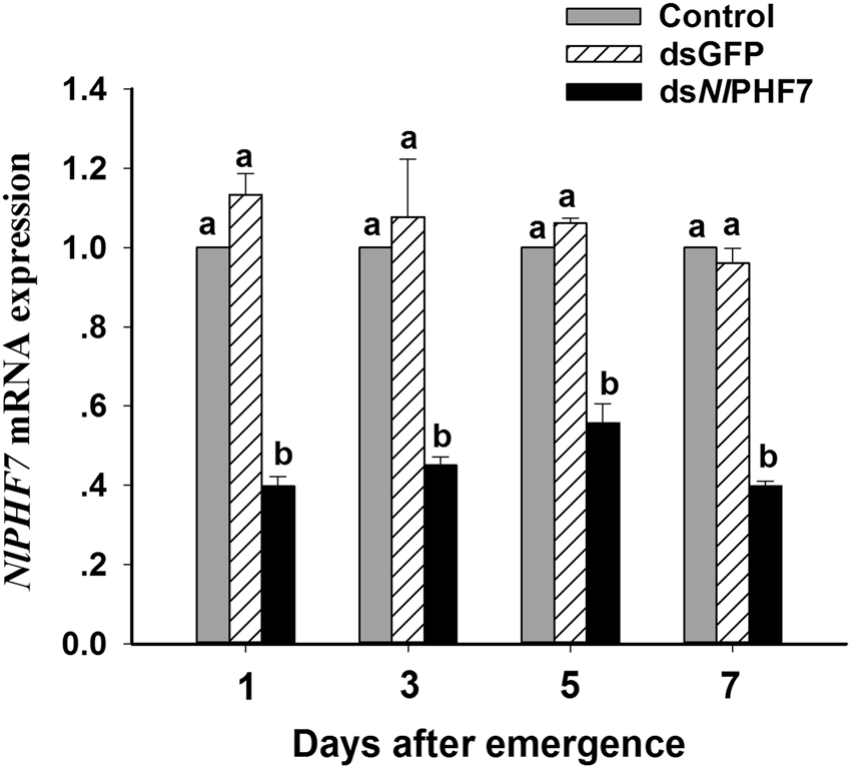
The influence of dietary ds*Nl*PHF7 on expression of the target gene during 1, 3, 5 and 7 days following adult male emergence. *NlPHF7* expression value of untreated males was converted to 1. The histogram bars show mean relative gene expression (n = three independent biological replicates) and the error bars represent standard deviation (t-test, *P* < 0.05). Gene expression was normalized using actin-1 as reference gene.

Figure 1 showed that *NlPHF7* expression level in the adult males emerged from the 3^rd^ instar nymphs fed with dietary ds*Nl*PHF7 was significantly down-regulated from 1, 3, 5 and 7 days post-emergence (DPE) (*F* = 186.1, df = 2, 24, *P* = 0.0001). Dietary ds*Nl*PHF7 treatments led to reduced *NlPHF7* expression in males, approximately by 44 ~ 60% compared to untreated control, and by 48% ~ 65% compared to dsGFP control at 1, 3, 5 and 7 DPE, respectively. However, no significant differences on the average of *NlPHF7* expression levels in the dsRNA-treated adult males between 1, 3, 5, and 7 DPE were observed (*F* = 1.1, df = 3, 24, *P* = 0.40) (Fig. 1). In addition, no remarkable interaction effects existed between DPE and dsRNA treatments in terms of the average expression levels of *NlPHF7* (*F* = 1.4, df = 6, 24, *P* = 0.25).

### Tissue-specific expression of the *NlPHF7* gene

**Figure 2:**
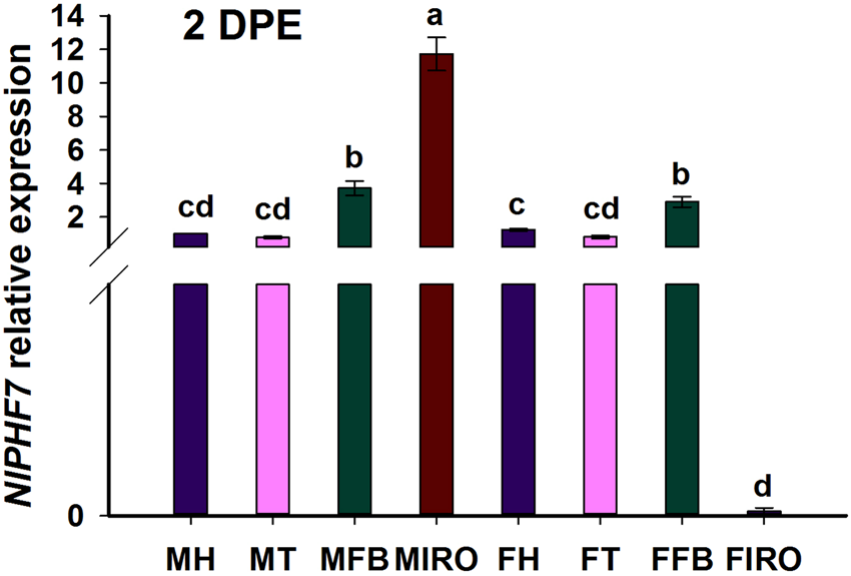
Expression of *NlPHF7* in various tissues of brachypterous adults (females or males) was determined by qPCR. MH: male head; MT: male thoracic; MFB: male fat body; MIRO: male internal reproductive organ; FH: female head; FT: female thoracic; FFB: female fat body; FIRO: internal reproductive organ. The expression value of male head was converted to 1. The mRNA level was normalized relative to actin-1 transcript. Histogram bars represent the mean values ± SE (n = three replicates). Histogram bars annotated with the same letter are not significantly different (t-test, *p* < 0.05).

To investigate the tissue-specific expression of *NlPHF7*, total RNA was isolated from male heads (MH), male thoraxes (MT), male fat bodies (MFB), male internal reproductive organ (MIRO), female heads (FH), female thoraxes (FT), female fat bodies (FFB) and female internal reproductive organ (FIRO) for qPCR analysis. Expression values of *NlPHF7* in MFB, MIRO, and FFB were significant higher when compared with that in MH, increasing by 2.7-, 10.7-, and 1.9-fold at 2 DPE, respectively, while no significant differences in *NlPHF7* levels between MFB and FFB tissues were observed (Fig. 2). However, *NlPH7* was barely expressed in FIRO (Fig. 2).

### Influence of dietary ds*Nl*PHF7 on adult males

**Figure 3:**
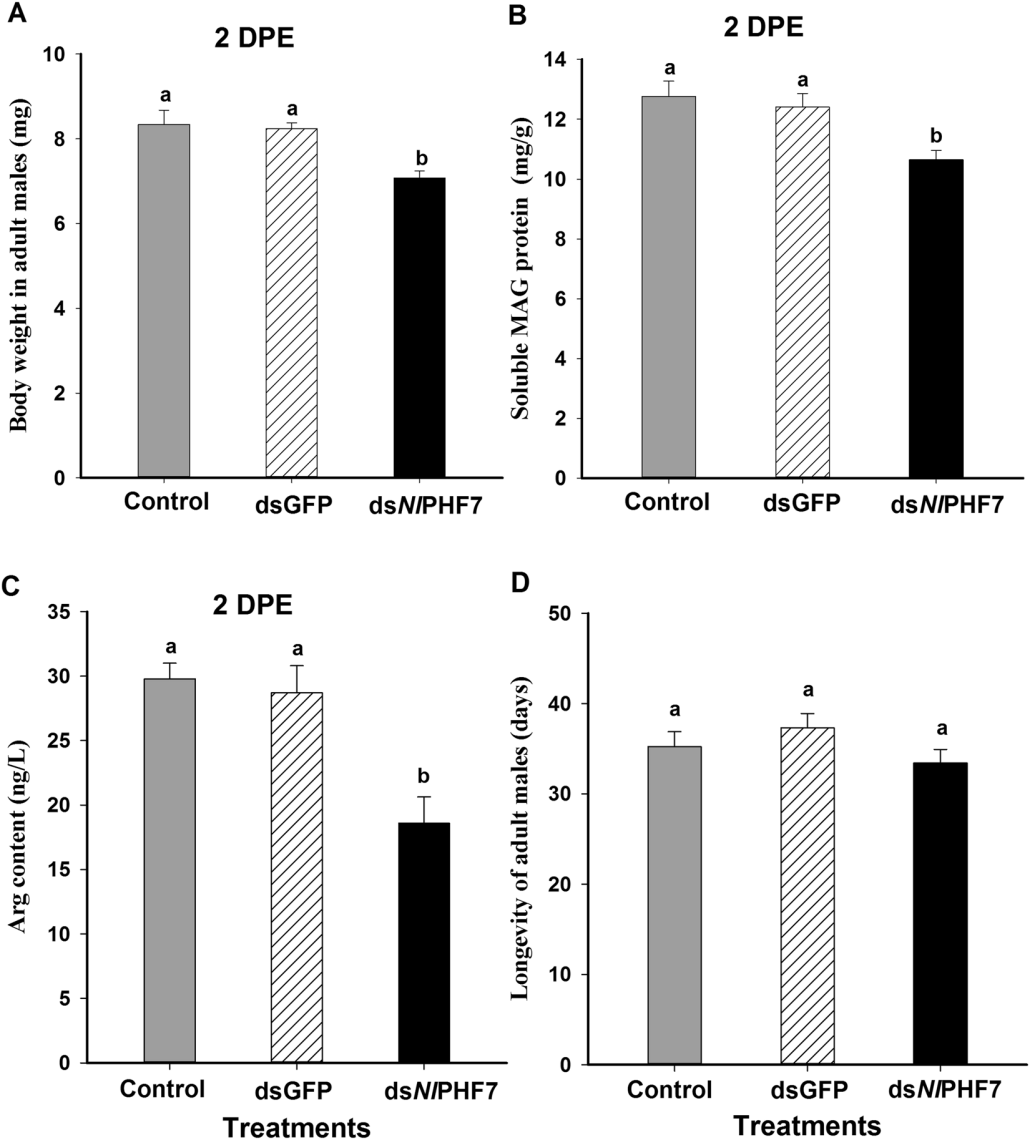
The effect of dietary ds*Nl*PHF7 on adult males. Panel (A): The histogram bars show mean body weight (n = five independent biological replicates (10 males/replicate). Panel (B): The histogram bars show mean soluble MAG protein content ± S.E (mg/g tissue, n = 9 independent biological replicates) at 2 DPE. Panel (C): The histogram bars show mean arginine content (ng/L) at 2 DPE. Panel (D): The histogram bars show mean longevity ± S.E (n = 23 independent biological replicates). Histogram bars annotated with the same letters are not significantly different (t-test, *p* < 0.05).

Adult males emerged from the 3^rd^ instar nymphs fed with dietary ds*Nl*PHF7 significantly decreased body weight and contents of soluble MAG protein and arginine at 2 DPE (*F* = 46.6, df = 2, 14, *P* = 0.0001 for body weight; *F* = 25.6, df = 2, 26, *P* = 0.001 for soluble MAG protein; *F* = 57.0, df = 2, 14, *P* = 0.0001 for arginine content) (Fig. 3). Dietary ds*Nl*PHF7 treatment in nymphs led to significantly reduced adult male body weight, down by 15% (from 8.3 mg to 7.1 mg) compared to untreated controls and by 13% (from 8.2 mg to 7.1 mg) relative to dsGFP controls (Fig. 3A). We also recorded reduction of soluble MAG protein content in treated males, down by 17% (from 12.8 mg/g to 10.6 mg/g) compared to untreated controls and by 14% (from 12.4 mg/g to 10.6 mg/g) compared to dsGFP controls (Fig. 3B). Dietary ds*Nl*PHF7 treatment in nymphs reduced arginine content in the resulting adult males, down by 38% (from 29.8 ng/L to18.6 ng/L) compared to untreated controls and by 35% (from 28.7 ng/L to 18.6 ng/L) relative to dsGFP controls (Fig. 3C). However, no significant impact on adult male longevity (*F* = 1.5, df = 2, 68, *P = *0.23) was recorded (Fig. 3D).

### Influence of dietary ds*Nl*PHF7 treated males on adult females via mating

**Figure 4:**
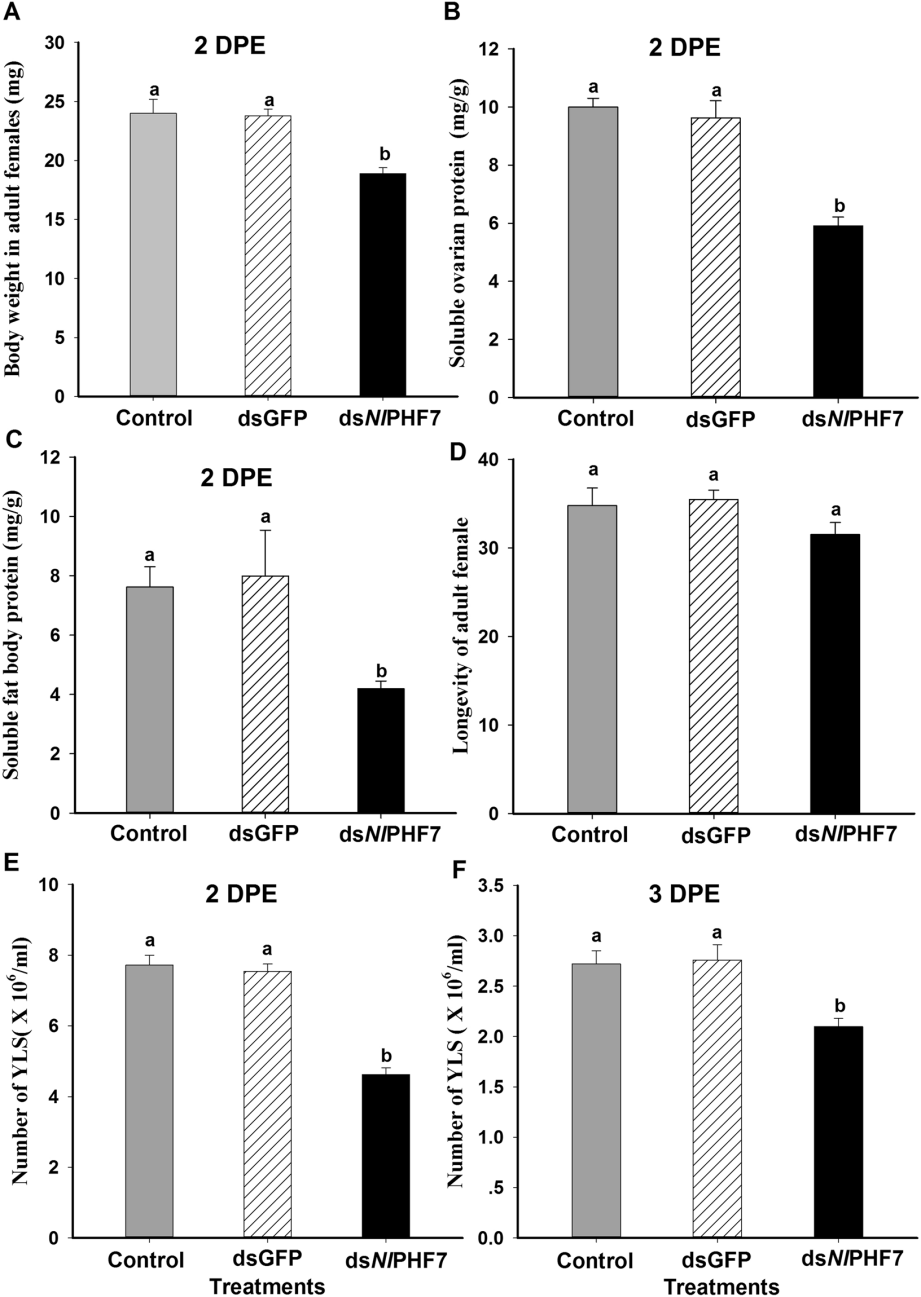
Influence of dietary ds*Nl*PHF7 of adult males on adult females via mating. Panel (A): The histogram bars show mean body weight (n = five independent biological replicates (10 males/replicate). Panel (B): Mean soluble ovarian protein content ± SE (mg/g) at 2 DPE. Panel (C): Mean soluble fat body protein content ± SE (mg/g) at 2 DPE. Panel (D): The histogram bars show mean longevity ± SE (n = 23 independent biological replicates). Panels (E and F): the histogram bars show mean numbers ± SE of YLS at 2 DPE and 3 DPE. Histogram bars annotated with the same letter are not significantly different (t-test, *p* < 0.05).

When adult males emerged from the 3^rd^ instar nymphs fed with dietary ds*Nl*PHF7-mated with the control female, the mating led to significantly reduced body weight and contents of soluble ovarian protein and soluble fat body protein in adult females at 2 DPE (*F* = 63.3, df = 2, 14, *P* = 0.0001 for body weight; *F* = 28.8, df = 2, 26, *P* = 0.0001 for soluble ovarian protein; *F* = 40.7, df = 2, 26, *P* = 0.0001 for soluble fat body protein) (Fig. 4A–C). The mating led to reduced body weight in females, down by 21% (from 24.0 mg/g to 18.9 mg/g) compared to untreated control males mated with the control females and by 20% (from 23.8 mg/mg to 18.9 mg/g) compared to dsGFP control males mated with the control females (Fig. 4A). Suppression of *NlPHF7* expression in males mated with the control females reduced soluble ovarian protein content in females, down by 41% (from 10.0 mg/g to 5.9 mg/g) compared to untreated control males mated with the control females and by 39% (from 9.6 mg/g to 5.9 mg/g) compared to dsGFP control males with the control females (Fig. 4B). We also recorded similar reductions in soluble fat body content in females, down by about 45% (from 7.6 mg/g to 4.2 mg/g) compared to untreated control males with the control females and by 47% (from 8.0 mg/g to 4.2 mg/g) compared to dsGFP controls males with the control females (Fig. 4C), but there was no impact of ds*Nl*PHF7**-**treated males on longevity of adult females via mating (*F* = 1.9, df = 2, 68, *P = *0.15) (Fig. 4D).

Control females mated with ds*Nl*PHF7-treated males significantly decreased YPS abundance in fat bodies of adult females at 2 and 3 DPE (*F = *44.4, df = 2, 14, *P = *0.0001 for 2DPE (Fig. 4E); *F = *289.3, df = 2, 14, *P* = 0.0001 for 3 DPE (Fig. 4F), down by 40% (from 7.72 × 10^6^/mL to 4.62 × 10^6^/mL) and by 30% (from 2.72 × 10^6^/mL to 2.10 × 10^6^/mL) at 2 and 3 DPE, respectively, compared to untreated control males mated with the control females; by 38% (from 7.54 × 10^6^/mL to 4.62 × 10^6^/mL) and 24% (from 2.76 × 10^6^/mL to 2.10 × 10^6^/mL) at 2 and 3 DPE relative to dsGFP males mated with the control females, respectively.

### Influence of dietary ds*Nl*PHF7 treated males on reproduction of females via mating

**Figure 5:**
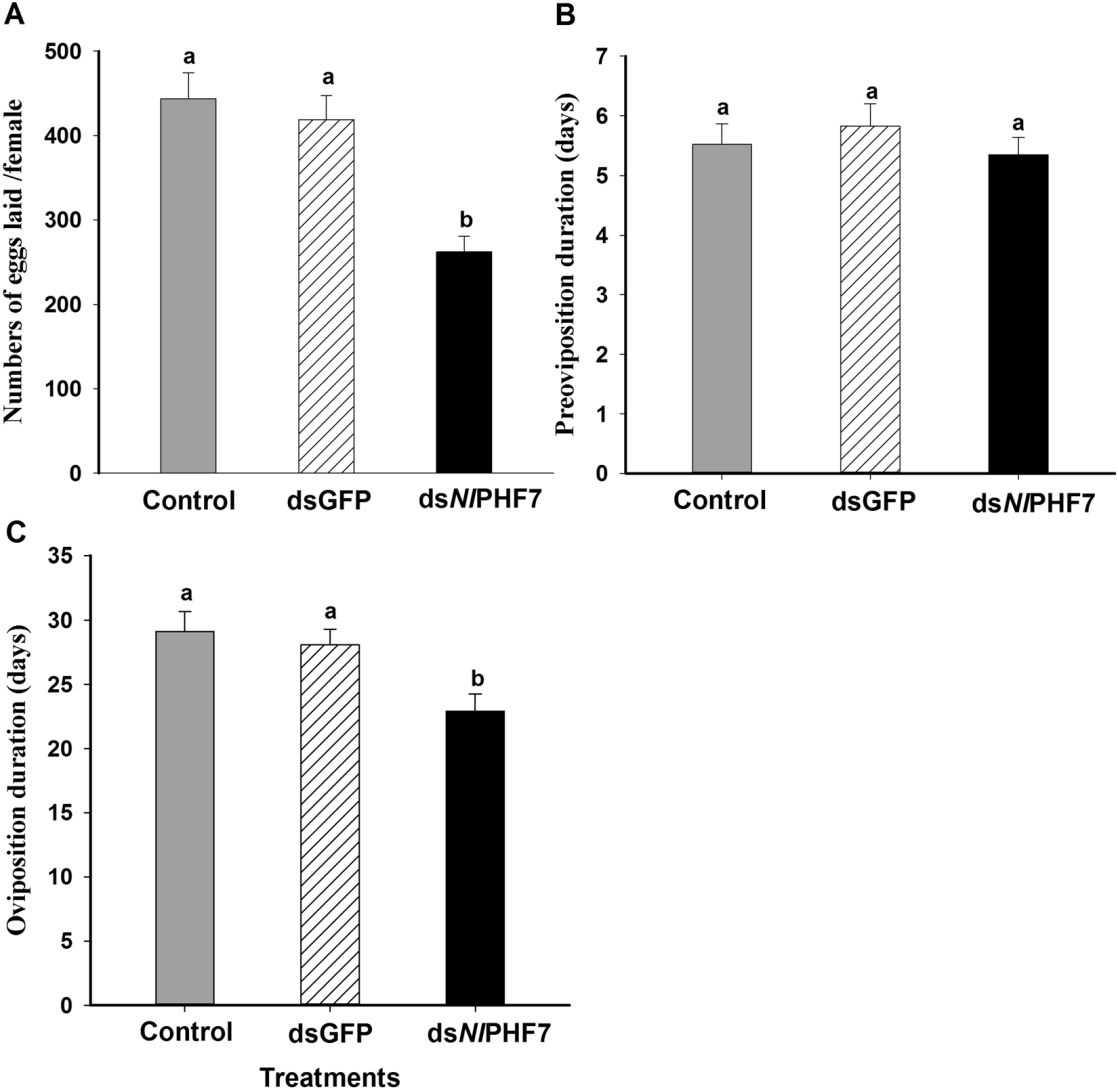
Influence of ds*Nl*PHF7 treated males on reproduction of female via mating. Panel (A): The ds*Nl*PHF7 treatments led to reduced fecundity. Panel (B): The dietary ds*Nl*PHF7 treatments did not influence pre-oviposition periods. Panel (C): The ds*Nl*PHF7 treatments led to decreased oviposition periods. Histogram bars represent mean numbers of eggs laid (Panel (A)) ± S.E or the number of days (Panel (B,C)). Histogram bars annotated with the same letter are not significantly different at *p* < 0.05.

Dietary ds*Nl*PHF7-treated males mated with the control female significantly decreased the number of eggs laid by adult females (*F* = 13.9, df = 2, 68, *P* = 0.0001), down by 40% (from 443 to 262 eggs/female) compared to untreated control males mated with the control females and by 37% (from 419 to 262 eggs/female) compared to dsGFP control males mated with the control females (Fig. 5A). The pre-oviposition period refers to the interval, in days, between adult emergence and the onset of first egg-laying. The preoviposition period was not affected by dietary ds*Nl*PHF7-treated males mated with the control females in adult females (*F* = 0.5, df = 2, 68, *P* = 0.6) (Fig. 5B). However, a shortened oviposition period was found in adult females (*F* = 5.9,df = 2, 68, *P* = 0.004), down by 21% (from 29.1 days to 22.9 days) compared to untreated control males mated with the control females and by 18% (from 28.0 days to 22.9 days) compared to dsGFP control males mated with the control females (Fig. 5C).

### Dietary ds*Nl*PHF7-treated males led to malformed internal reproductive organ

**Figure 6:**
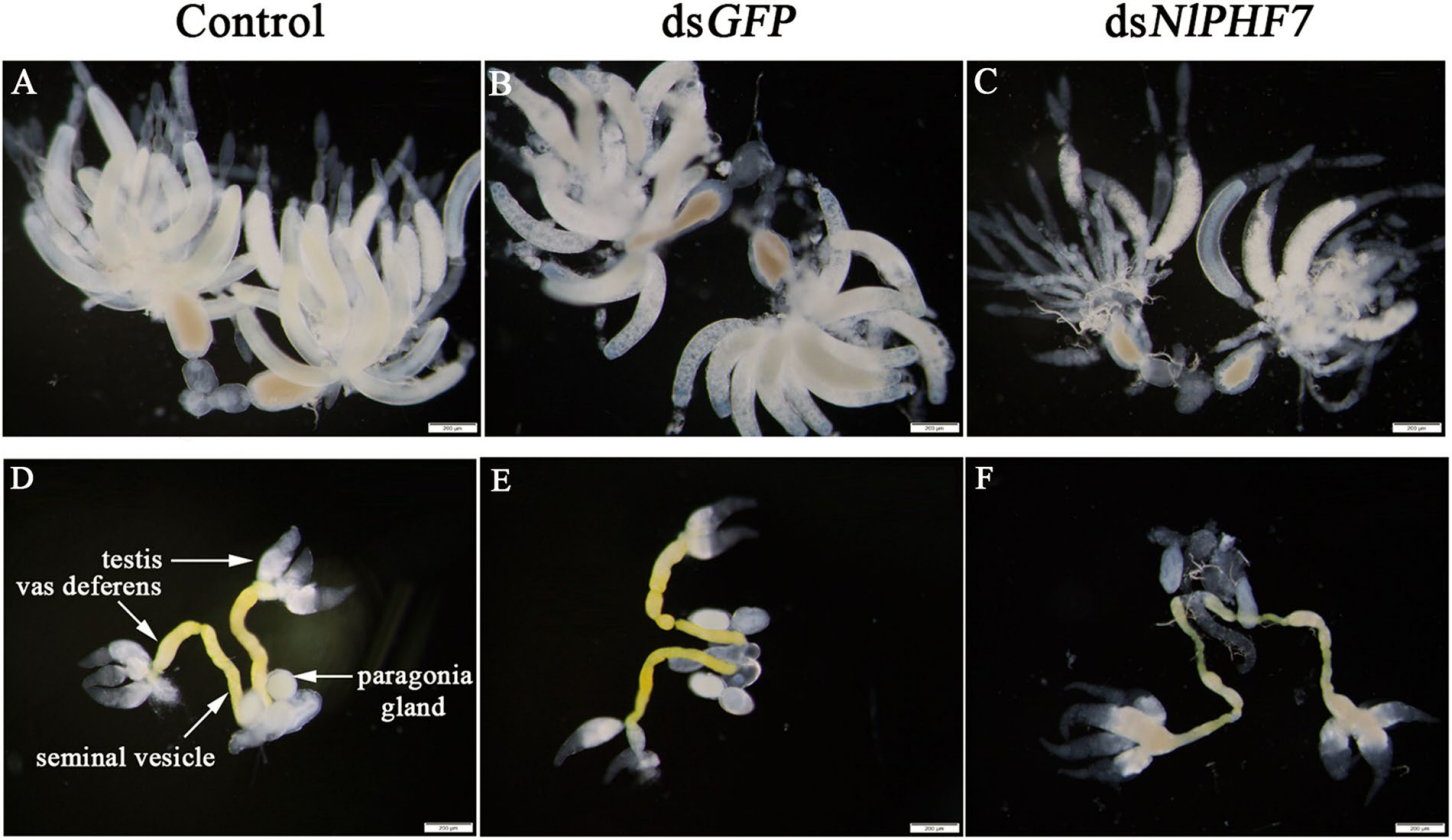
Dietary ds*Nl*PHF7 led to malformed reproductive systems at 2 DPE. The third instar nymphs were treated with dietary ds*Nl*PHF7. Panels (A–C): Reproductive tracts were isolated from females and photographed using an OLYMPUS SZX16 microscope. The reduced sizes of ovaries were noticed. Panels (D–F): Reproductive tracts were isolated from males and photographed using an OLYMPUS SZX16 microscope. The reduced sizes of the vas deferens, testis, and seminal vesicle were observed.

The external morphology of ovaries prepared from females (control-♀) mated with experimental males (dsNlPH7-♂ or dsGFP-♂), was also influenced by dietary dsRNA construct. Observation reveals that the ovarioles in the control females mated with untreated males (Fig. 6A) or with dsGFP-treated males (Fig. 6B), had one or two fully developed banana-shaped oocytes at 2DPE. However, control females mated with males exposed to dietary ds*Nl*PHF7 during the nymph stage resulted in undeveloped ovaries and severely inhibited oocyte growth in the ovaries (Fig. 6C). No fully developed oocytes were observed at 2DPE in the ds*Nl*PHF7-treated group (Fig. 6C).

Compared to untreated males (Fig. 6D) and dsGFP-treated males (Fig. 6E), dietary ds*Nl*PHF7 treatment led to significant malformation of vas deferens, seminal vesicle, and influenced testis development in males internal reproductive organ (MIRO) at 2 DPE (Fig. 6F), but no externally visible effect on paragonia glands was observed (Fig. 6F).

### Dietary ds*Nl*PHF7-treated males led to reduced *Nlvg* mRNA expression via mating

**Figure 7:**
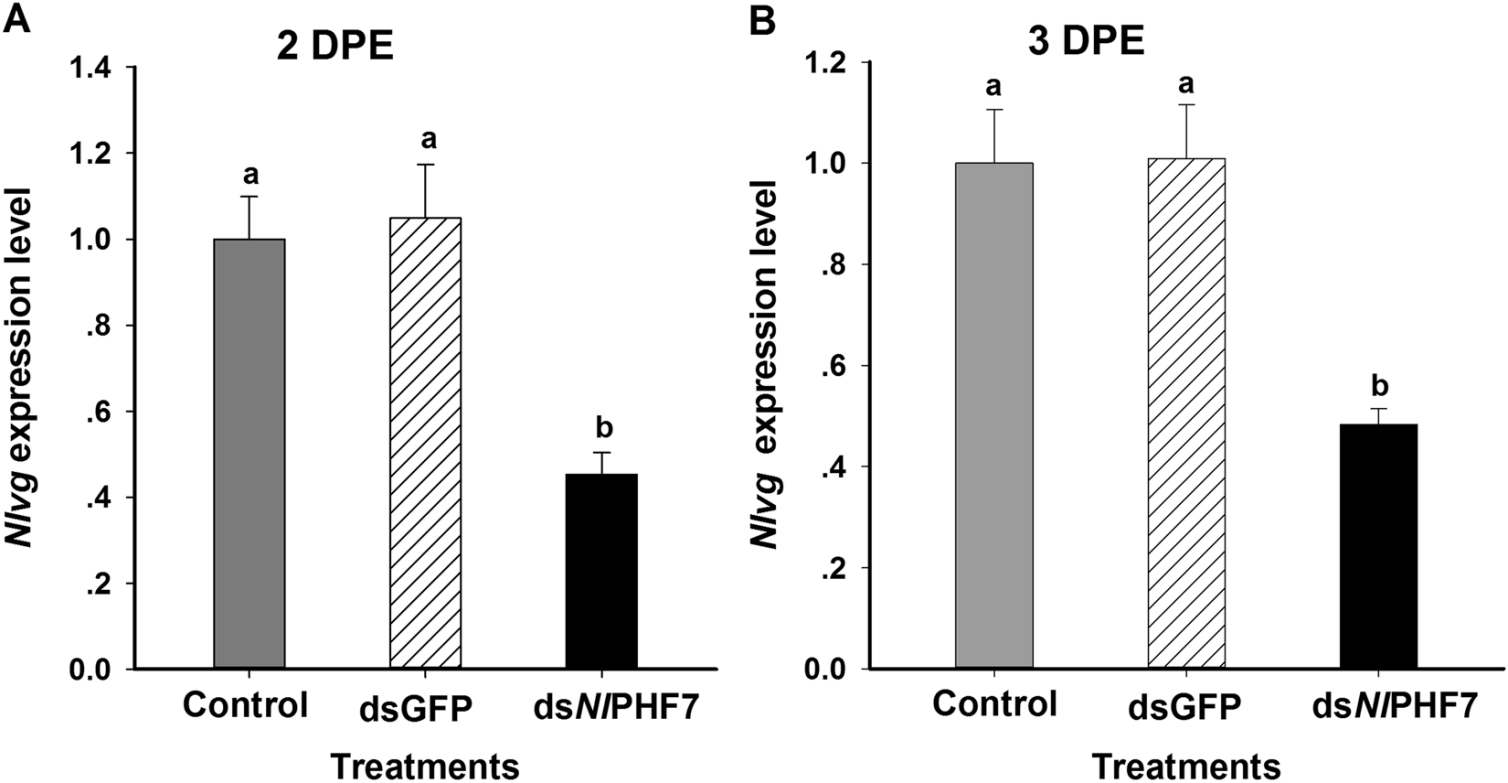
Control females mated with the dietary ds*Nl*PHF7 males led to decreased *Nlvg* mRNA expression at 2 DPE (**A**) and 3 DPE (**B**). Values were normalized relative to the reference gene, actin-1. Histogram bars represent the mean values ± SE (n = three replicates). Histogram bars annotated with the same letter are not significantly different (t-test, *p* < 0.05).

We examined the expression level of *Nlvg* mRNA in the control females mated with ds*NlPHF7*-treated and dsGFP-treated males (Fig. 7). The control females mated with males exposed to dietary ds*Nl*PHF7 or dsGFP led to significantly down-regulated *Nlvg* expression at 2 DPE (*F* = 53.9, df = 2, 8, *P* = 0.0001), down by 55% compared to the control females mated with untreated males group and by 57% compared to the control females mated with dsGFP-treated males at 2DPE (Fig. 7A). We also recorded similar reduction in *Nlvg* expression at 3 DPE (*F* = 66.2, df = 2, 8, *P* = 0.0001), down by 51% compared to the control females mated with untreated males and by 52% compared to the control females mated with dsGFP-treated males at 3 DPE (Fig. 7B).

### Dietary ds*Nl*PHF7 treatments males led to reduced offspring and hatching rate

Table 1 showed that dietary ds*Nl*PHF7 significantly reduced the number of offsping and hatching rate when the control females mated with ds*Nl*PHF7-treated males (*F* = 31.1, df = 3, 19, *P* = 0.0001 for number of offspring; *F* = 36.1, df = 3, 19, *P* = 0.0001 for hatching rate) (Table 1). The dietary ds*Nl*PHF7-treated males mated with the control females led to reduced number of offspring, down by 66% (from 422 to 143) compared to untreated control males mated with the control females and by 67% (from 438 to 143) relative to dsGFP-treated males mated with the control females. Similarly, the control females mated with ds*Nl*PHF7-treated males reduced egg hatching rate, down by 18% (from 0.93 to 0.76) compared to untreated control males mated with the control females and by 19% (from 0.94 to 0.76) relative to dsGFP-treated males mated with the control females. However, the dietary ds*Nl*PHF7 treatments did not influence gender ratio of offspring (Table 1).

**Table 1:** Influence of dietary ds*Nl*PHF7-treated males mated with the control females on the number of offspring, hatching rate, and gender ratio.

Treatment	Number of offspring	Hatching rate	Gender ratio	PGI(N1/N0)
Control	422.0 ± 73.3a	0.93 ± 0.02a	1.90 ± 0.68a	105.5
dsGFP	438.8 ± 89.7a	0.94 ± 0.01a	1.91 ± 0.60a	109.7
dsNlPHF7	143.0 ± 16.7b	0.76 ± 0.05b	2.47 ± 0.19a	35.8

^*^Means ± SE of five replicates. Means within columns followed by different letters are significantly different at the 5% level (*p* < 0.05, PLSD test). Control group: control-♀ × dsNlPHF7-♂; dsGFP group: control-♀ × dsGFP-♂; ds*Nl*PHF7 group: control-♀ × ds*Nl*PHF7-♂.

## Discussion

The data reported in this paper strongly support our hypothesis that suppressing *NlPHF7* expression in males influences the reproductive physiology of female partners via mating, which reduces BPH population growth in agroecosystems. Our data show the following five conclusions: 1) *Nl*PHF7 was highly expressed in MFB, MIRO and FFB tissues at 2 DPE; 2) dietary ds*Nl*PHF7 treatments reduced *NlPHF7* expression, body weight, soluble MAG protein contents, arginine contents, and malformed IRO in males; 3) similar treatments with experimental males reduced numbers of YLS, ovarian development, soluble ovarian protein contents and soluble fat body protein contents in their untreated female mating partners; 4) dietary ds*Nl*PHF7 treatments in males altered female reproductive biology, including shortened oviposition periods and reduced *Nlvg* expression and egg laying; 5) Suppression of *NlPHF7* level reduced the number of offspring and egg hatching rate. However, the treatments did not influence adult longevity and preoviposition periods. Taken together, these data indicate that suppressing expression of *NlPHF7* in males strongly altered some, but not all, aspects of female reproductive biology. The significance of this finding lies in a direct demonstration of the influence of a single male-derived protein on female partners via mating.

Spermatogenesis is a highly regulated complex process, involving both intrinsic and extrinsic regulators, as well as interaction between germ cells and sertoli cells^15^. The intrinsic regulation of gene expression in spermatogenesis occurs at three phases: transcription, translation and post-translation. Spermatogenesis is further subjected to secondary regulation by endocrine, paracrine, and autocrine signals transmitted indirectly through surrounding somatic cells including Sertoli cell^16^. Defective spermatogenesis may result from systemic disease, malnutrition, endocrinological disorders, genetic defects, anatomical obstruction, infections, and environmental toxins^17^. NYD-SP6 (PHF7) might activate transcription through PHD zinc finger, regulating potential protein likely responsible for cell proliferation including germ cell development^11^. Many nuclear transcription factors, which are important for germ cell development, have been reported to contain PHD finger domain, which is localized in nucleus and implicated in transcriptional regulation^18, 19^. *Nl*PHF7 is expressed throughout the first seven days of male adulthood, allowing the protein to sexually transfer to females during each mating. The current results showed that dietary ds*Nl*PHF7 treatment resulted in malformation of vas deferens, seminal vesicle, and retardation of testis growth in MIRO at 2 DPE. We speculate that knockdown *NlPHF7* influences spermatogenesis transcription phases, and also might further influence cognate gene expression in spermatogenesis, which influences male spermatogenesis phases. In adult males of *Ephestiacautella*, a greater size or a heavier weight has been used as an indicator of “good quality”, regarded as a possession of optimal genes and a larger sperm supply^20, 21^. Males that mate with heavy or average-sized *C. jactatana* females may sire more offspring, which have a higher rate of survival^22^. Our current results showed that dietary ds*Nl*PHF7 treatment resulted not only in malformation of vas deferens, seminal vesicle, and retardation of testis growth in MIRO at 2 DPE, but also decreased male body weight and female body weight, and reduced number of offspring and hatching rate of eggs via mating. Therefore, our findings imply that *Nl*PHF7 plays an important role in BPH reproduction and population growth.

PHF7 is expressed in both males and females of *D. melanogaster*, with a predominant expression in MIRO. Loss-of-function of PHF7 mutation affected spermatogenesis, but not oogenesis in *D. melanogaster*, indicating that the PHF7 gene is important for fertility in males but not in females^12^. Therefore, PHF7 is called the male reproductive gene. Silencing *NlPHF7* might reduce the expression of cognate gene and inhibit the development of vas deferens, seminal vesicle, and testis, which accordingly led to reduced MAG proteins in males. It has been elucidated that the male paternity share of female’s progeny are influenced by many factors such as nutrient status^23^, male dominance^24^ and MAG proteins^25^. In *D. melanogaster*, MAG proteins mediate a variety of effects that benefit males, including the stimulation of female egg protection and receptivity reduction after mating^26–28^. Likewise, MAG protein with the treated males increase oviposition rate and sexual receptivity and decrease life span in female crickets^29^. Many insect studies have also demonstrated that male accessory gland peptides regulate female reproductive performance via mating. For example, the adult male MAG proteins of *D. melanogaster* synthesize and secrete a peptide, thus stimulating oviposition^30^. MAG proteins regulate spermatophore formation, egg maturation and oviposition in *Heliothis virescens* ^31^. Also, MAG proteins can influence reproductive behaviors such as remating, ovulation, and egg-laying^32, 33^. Previous studies have demonstrated that insecticide treatments enhanced BPH male accessory gland (MAG) protein content^9^. Compared to copulation with untreated males, females mated with insecticide-treated males increased fecundity, registered as increased numbers of eggs laid^9^. *NlPHF7* gene expression level in tzp-treated male *N. lugens* was significantly higher than that of untreated control^10^. Similarly recorded, dietary ds*Nl*PHF7 treatment led to reduction of arginine content. Arginine, a necessary amino acid for sperm, is associated with spermatogenesis. Arginine is a precursor in the synthesis of nitric oxide (NO). NO promotes spermatogenesis and enhances sperm vigor and sperm insemination ability^34, 35^. Therefore, we infer that dietary ds*Nl*PHF7 might influence sperm motility, sperm counts, and spermatogenesis in adult males. Additionally, fewer MAG proteins or nutrition substances transferred to adult females via mating cause fewer eggs to be laid.

Surprisingly, *NlPHF7* suppression in males by RNAi significantly influenced female reproductive biologys, resulting in decreased protein content in fat bodies and ovaries and reduced number of egg-laid by adult females. Zeng *et al*.^36^ reported that egg production showed a positive correlation with vitellogenin content of fat body in *Helioths virescens*. The reduced protein contents can disrupt BPH biology, including production of yolk proteins. Vitellogenins are produced in fat bodies and the reduced fat body protein contents may impose a limitation on the ability to produce vitellogenin, which can restrict ovarian development and reproduction. The relative expression of *Nlvg* encoding Vg was reduced in females after mating with ds*Nl*PHF7-treated males as shown in the present study. The previous results demonstrated that MAG protein of tzp-treated males was significantly increased compared to untreated control and JH III titer was increased as well^9, 37^, thus stimulating female fecundity via mating^9^. Likewise, proteins in fat body and ovary of females mated with tzp-treated males were significantly increased compared to females mated with untreated males^37^. The current data also showed that suppression of *NlPHF7* reduced BPH population growth and hatching rate of eggs-laid. We infer that dietary ds*Nl*PHF7-treated males might transfer fewer nutrition substances or hormonal substances to females via mating, resulting in decreased *Vg* expression and Vg synthesis as well as egg-laying in adult females.

Dietary ds*Nl*PHF7 treatments led to reduced YLS abundance in adult females at 2 DPE and 3 DPE via mating. The previous investigation has shed light on a relationship between YLS and amino acid requirements in BPH^38, 39^. The BPH requirements of essential amino acids were closely related to the abundance of YLS^40^. Artificial reduction of the symbionts abundance delayed growth and decreased survival rate, adult emergence rate, body weight, and fecundity^41^. Symbiotic bacteria in pea aphids also significantly affected amino acid metabolism^40^. In terms of the physiological role, the YLS provides essential amino acids and possible proteins required for host embryo formation and post-embryonic development^42^. The symbionts are essential to meet nutritional amino acid needs of hosts for production of a healthy complement of proteins. Therefore, we infer that suppression *NlPHF7* expression in males might transfer fewer accessory gland proteins (including nutrition substances) to females via mating, and result in less symbiont maintenance in females, and influence female reproduction. In the present study, we found that YLS abundance of control females mated with dietary ds*Nl*PHF7-treated males at 3 DPE was significantly lower than that in those mated with ds*Nl*PHF7-treated males at 2 DPE. The number of symbiotic bacteria increased significantly with growth of nymphs, and the density of *Wolbachia* in the adult population of Guangxi *N. lugens* was greatly reduced with the increase of age, which is consistent with our previous experimental results^43^. YLS abundance can be influenced by many factors, which supply insect energy and nutrition. Changes of fat and glycogen contents in fat body also affect the number of YLS in *N. lugens* ^44^. Environmental factors are considered to influence the density of YLS in hosts. Temperature is the most important influence factor, and its interaction with host symbiosis has already been reported^45^.

In summary, *Nl*PHF7 in males plays an important role in regulating female fecundity, egg hatching rate, and population growth via mating. It exerts profound molecular and biochemical effects on production of Vg and likely other proteins, and these effects can be indirectly registered at the levels of organismal reproduction and BPH population dynamics. This study not only provides evolutionary insights into reproductive strategies, but also facilitates the development of novel means for controlling insect pests via identifying effective target genes in males.

## Materials and Methods

### Rice variety and culture

Rice (*Oryza sativa* L.) variety Ninjing 4 (japonica rice, commonly grown in Jiangu province) was used in all experiments. Seeds were sown outdoors in cement tanks (height 60 cm, width 100 cm, and length 200 cm) containing standard rice-growing soil. When seedlings reached the six-leaf stage, they were transplanted into 16 cm diameter plastic pots containing four hills per pot, three plants per hill and used for experiments at the tillering stage.

### Insect culture

BPHs were obtained from a laboratory population maintained in a greenhouse under our standard conditions (26 ± 2 °C, with 70–80% humidity and a 16 L: 8D photoperiod) at Yangzhou University. The insect colony was originally obtained from the China National Rice Research Institute (Hangzhou, China). Before the experiments started, the colony was allowed to reproduce for two generations in cement tanks (60 × 100 × 200 cm) under natural conditions in Yangzhou.

### dsRNA synthesis

We designed gene-specific ds*Nl*PHF7 primers and amplified a 310-bp (388–707 bp) *NlPHF7* cDNA fragment using forward and reverse primers containing the T7 primer sequence at the 5′ ends (Table 2). The amplification program was 35 cycles of 95 °C for 40 s, 58 °C for 40 s and 72 °C for 1 min, with a final extension step of 72 °C for 10 min. The sequence was verified by sequencing (Invitrogen, Shanghai, China). We used the GFP gene (ACY56286; provided by Zhang Chuan-xi, Institute of Insect Sciences, Zhejiang University) as control dsRNA and amplified a 688 bp fragment using primers listed in Table 2. For *NlPHF7* and the control GFP gene, we used the T7 RiboMAX^TM^ Express RNAi System (Promega, Sunnyvale, CA) for dsRNA synthesis, following the Promega instructions. We generated sense and antisense dsRNAs in separate 20 µL reaction volumes. The dsRNAs were annealed by mixing and incubating at 70 °C for 10 min, and then cooling to room temperature over 20 min. A 2 µL RNase A solution (4 mg/ml) and 2 µL RNase-free DNase (1 u/µL) were added to the reaction tube and incubated in a 37 °C water bath for 30 min. The dsRNA was precipitated by adding 110 µL 95% ethanol and 4.4 µL 3 M sodium acetate (pH 5.2), then washed with 0.5 mL 70% ethanol and dried at room temperature. The dried product was dissolved in 50 µL nuclease-free water. The purified dsRNAs were quantified by spectroscopy. To deliver dsRNA into BPH, nymphs were reared on an artificial diet amended with dsRNA^46^, with some modifications to the rearing protocol. Previous results indicated that dsRNA feeding led to rapid and significant reduction in expression levels of BPH genes^47^. We used glass cylinders (15.0 × 2.5 cm diameter) as feeding chambers, with four dsRNA concentrations, 0.125, 0.075, 0.05, and 0.025 μg/μL. The dsRNA solution (final concentration, 0.05 μg/μL diet determined from the concentration response assay just mentioned, was used in all dsRNA assays. The artificial diet (20 μL) was held between two layers of stretched Parafilm M membrane enclosed at the two open ends of the chamber (the diet capsule). The diet capsule was replaced every second day. The cylinders were covered with a piece of black cotton cloth, but the two ends with the artificial diet were exposed to light. Insects fed on the diets by puncturing the inner Parafilm M membrane of the diet capsule. Experimental insects were transferred into chambers and maintained on artificial diets for one day before initiation of the assays. Twenty 3^rd^ instar individuals were transferred into each chamber, and three chambers were used to create three independent biological replicates. The rearing experiments were carried out in a humidified growth cabinet at 26 ± 2 °C, 90% RH and a 16 L: 8D photoperiod. Mortality was recorded every other day.

**Table 2:** PCR primers used in this study.

Primers	Primer sequence	Product size
For real-time PCR		
Q-*NlPHF7*-F	GATGGTCGAAGATTAAGACT (Tm = 53.7 °C)	114 bp
Q-*NlPHF7*-R	AACAGTAGAAGCGGGAAT (Tm = 52.7 °C)	
Q-*Nlvg*-F	GTGGCTCGTTCAAGGTTATGG (Tm = 58.0 °C)	200 bp
Q-*Nlvg*-R	GCAATCTCTGGGTGCTGTTG (Tm = 60.2 °C)	
Actin-F	TGCGTGACATCAAGGAGAAGC (Tm = 60.0 °C)	186 bp
Actin-R	CCATACCCAAGAAGGAAGGCT (Tm = 60.0 °C)	
For dsRNA synthesis		
* NlPHF7*-F	TAATACGACTCACTATAGGG (T7 promoter)	310 bp
	CCGAAGAACATAGTGGATG (Tm = 66.4 °C)	
* NlPHF7*-R	TAATACGACTCACTATAGGG (T7 promoter) CAGTGTCACAGTGCTGGTAG (Tm = 68.4 °C)	
For dsRNA synthesis		
* NlGFP*-F	TAATACGACTCACTATAGGG (T7 promoter)	688 bp
	AAGGGCGAGGAGCTGTTCACCG (Tm = 60 °C)	
* NlGFP*-R	TAATACGACTCACTATAGGG (T7 protomer)	
	CAGCAGGACCATGTGATCGCGC (Tm = 56 °C)	

### Influence of dietary dsRNA on biological performance parameters

We determined the effects of ds*NlPHF7* treatments on selected biological performance parameters. In preliminary experiments, we exposed second instar nymphs to the dsRNA construct, which led to over 95% mortality. Thereafter, we transferred 3^rd^ instar nymphs to capsules containing dsRNA-laced diet. When the nymphs reached fifth (final) instar (about 8 days), they were collected, individually transferred into a glass jar (12 cm high × 10 cm), and reared on tillering rice plants under 26 ± 2 °C, RH 90%, and 16 L: 8D. One newly emerged, untreated female (feeding artificial diet, control-♀) mated with one newly emerged dsRNA-treated male (ds*Nl*PHF7-♂ or dsGFP-♂). We choose to assess many of the parameters at 2 DPE since the proteomic analysis of the *N. lugens* at 2 DPE showed that *Nl*PHF7 was significantly increased^4^. One hundred adult females and 100 adult males were collected separately at 2 days after mating. Fresh body weight, soluble ovary protein and soluble fat body protein content, contents of MAGs and arginine, and *NlPHF7* expression level were determined. Then we individually paired a newly-emerged untreated female (feeding artificial diet, control-♀) with a dsRNA-treated male (ds*Nl*PHF7-♂ or dsGFP-♂). Each pair (ds*Nl*PHF7-♂ × Control-♀ or dsGFP-♂ × Control-♀ or Control-♂ × Control-♀) was maintained in a glass jar (diameter 10 cm, height 12 cm) with rice seedlings under our standard conditions (26 ± 2 °C, RH 90%, and 16 L: 8D) for oviposition. Twenty-three copulating pairs were maintained to record duration of the pre-oviposition period, oviposition period, adult longevity, and fecundity for each pair. Rice stems were replaced daily during the pre-oviposition period, at two day intervals during the oviposition period and three day intervals during the female longevity period until the females died. The numbers of eggs laid on each rice stem were recorded under a light microscope. Fecundity of 23 mated pairs was evaluated by the average number of eggs laid.

### Protein Extraction and determinations

Protein was extracted from fat bodies and ovaries using a method similar to Gong *et al*.^48^. Individual adult females in all 15 females 2 days post-emergence (2 DPE) were dissected under a zoom stereomicroscope (model XTL20, Beijing Tech Instrument Co., Ltd., Beijing, China) in a cooled petri dish. Ovaries and fat bodies were removed and placed in separate, pre-weighed, ice-cold centrifuge tubes and then re-weighed using a Mettler-Toledo electronic balance (EC100 model; 1/10,000 *g* sensitivity). A proportional amount of NaCl solution (0.4 M NaCl: 1 M PMSF, v:v at a ratio of 20 mL NaCl solution to 1 g ovary or fat body) was added to the tube, homogenized on ice, and centrifuged at 16,000 × *g* at 4 °C for 20 min. The supernatant was collected after filtering the upper fat layer with glass fibers, placed at 4 °C overnight after adding ddH_2_O (1 supernatant: 10 ddH_2_O, v/v), and centrifuged again at 4,000 × *g* at 4 °C for 20 min. The protein sediment was dissolved with 1.5 ml pre-chilled 0.4 M NaCl solution after removing the supernatant. Protein from MAGs was extracted using the methods of Ge *et al*.^41^. Individual adult males in all 10males were dissected under a zoom-stereomicroscope in a cooled petri dish. MAGs were removed and placed in separate, pre-weighed, ice-cold Eppendorf tubes. To each tube, 600 µL of mixed solution (methanol/distilled water/acetic acid/ methyl thioethanol; 80:18:2:0.1; v:v:v:v) was added. The contents were homogenized on ice and centrifuged at 12,000* × g* at 4 °C for 10 min. The upper fat layer was removed to yield the supernatant. Four hundred µL of mixed solution was added to the sediment in the tube, which was then centrifuged again and the supernatant collected.

We followed Bradford^49^ to measure protein content using Coomassie Brilliant Blue R250 (Shanghai Chemical Agent Co., Ltd., Shanghai, China). A standard curve was established based on a BSA standard protein (Shanghai Biochemistry Research Institute, Shanghai, China). The absorbance at 595 nm was determined in a UV755 B spectrometer (Shanghai Precision Instrument Co., Ltd., Shanghai, China). The protein content in the sample solution was calculated according to the standard curve. Protein determinations were repeated nine times, with nine independent biological samples.

### Determination of arginine content

We followed the instruction from an insect arginine ELISA kit (Qiaodu biological technology Co., LTD, Shanghai, China) to measure arginine content in insect tissues using the double antibody sandwich ELISA assay. Each treatment and control group (including 10 males, respectively) 2 days post-emergence (2 DPE) were accurately weighed, 1 mL PBS (pH7.4) was added, then homogenized adequately by hands, centrifuged at 3,000 × g for 20 min. The supernatants were collected carefully and refrigerated at −20 °C. A total of 10 standard wells on the coated ELISA plates were used for the assay. 100 μL of standard substances and 50 μL of the standard dilution were respectively added to the first and second well, shacking adequately. Then 100 μL was taken from the each well, 50 μL of dilution was added to the third and fourth well, blended thoroughly. Subsequently, 50 μL of mixtures from the third and fourth well was removed, and 50 μL of mixtures each from the two wells were taken to the fifth and sixth well, mixed with 50ul of standard dilution, respectively. After that, 50 μL of mixtures from the fifth and sixth well and 50 μL of standard dilution were added to the seventh and eighth well, respectively, blended thoroughly. Similarly, 50 μL of mixtures from the seventh and eighth well and 50 μL of standard dilution were added to the ninth and tenth well, respectively, blended thoroughly. Finally, 50 μL of mixtures from the ninth and tenth well was removed (the final dilution, the sample volume of each well was 50 μL and concentrations were 12 U/L, 8U/L, 4 U/L, 2U/L, 1 U/L.). Forty μL of standard dilution and 10 μL of the samples (final dilution was 1:5) were added to the test wells on the coated ELISA plates. The samples were required to avoid touching the wall of wells, added to the bottom of the plates. We lightly shacked the plate to mix them well. The plates were divided into blank pore and sample wells. Plates were sealed with plate sealers, incubated at 37 °C for 30 min. The sealing film was peeled off carefully and solution was removed. The ELISA plate wells were dried, filled with washing liquid, allowed to stand for 30 s, the liquid was removed, which were repeated five times and pat-dried. Enzyme linked reagents (50 μL) were added to each well, with an exception of the blank pore. Plates were sealed with plate sealers, incubated at 37 °C for 30 min. The sealing film was peeled off carefully and solution was removed. The ELISA plate wells were dried, filled with washing liquid, allowed to sit for 30 s, the liquid was removed, which were repeated five times and pat-dried. Color developing agents A (50 μL) and B (50 μL) were added orderly in each well. The mixtures were gently shacked to get well mixed. The reaction was sheltered from light, kept at 37 °C for 15 min. The reaction ended up with adding 50 μL terminal liquid in each well (The color blue immediately changed to yellow). We used blank as a reference for the zero-setting and detected every pore’s absorbance (OD values) at the wavelength of 450 nm. This operation must be performed within 15 min after adding the terminal liquid. The standard curve was established with the concentration of standard substances as x-coordinate and OD values as the y-coordinate. The corresponding concentration of samples can be found in the standard curve according to its OD value. The sample concentration should be multiplied by dilution times. Each treatment and control was repeated five times.

### Observation of the number of yeast-like symbionts (YLS) in fat bodies

The procedure described in Noda^50^ was followed to measure the number of YLS of fat bodies with blood cell counter (0.01 mm, 1/400 mm^2^) (25*16 model, Shanghai Qiujing Biochemical Reagent Co., Shanghai, China). We transferred the 3^rd^ instar nymphs to capsules containing dsRNA-laced diet; at their fifth (final) instar (8 days), nymphs were individually transferred into a glass jar (12 cm high × 10 cm) and reared on rice plants at the tillering stage under 26 ± 2 °C and 16 L:8D. We paired individually paired a newly-emerged female (feeding artificial diet, ♀control) with a dsRNA-treated male (ds*Nl*PHF7-♂ or dsGFP-♂). Each pair (ds*Nl*PHF7-♂ × control-♀ or dsGFP-♂ × control-♀or control-♂ × control-♀) was maintained in a glass jar (diameter 10 cm, height 12 cm) with rice seedlings under our standard conditions (26 ± 2 °C, RH 90%, and 16 L: 8D) for observation of the number of YLS in fat bodies. Sixty uniform adult females were collected separately at 2 or 3 days after mating. This parameter was closely associated with insect energy and nutrition supply sites. We separately collected BPHs at 2 or 3 days after mating to evaluate the dynamic number of YLS in fat bodies at different DAE (day after emergence). Fat body was dissected from six adult females in treated (dsRNA-♂ × control-♀ and dsGFP-♂ × control-♀) or untreated control (control-♂ × control-♀) group and homogenized gently in 200 µl saline solution (0.9% NaCl). Homogenate (2 µL) was added to a blood cell counter (0.01mm, 1/400mm^2^) and the numbers of YLS were counted under microscope using a 5 point sampling method. The numbers of YLS were counted from 80 squares (unit, mm^2^) each time. Each treatment and control was replicated five times.

### Body weights

Ten females or males were used as a replicate at 2 DPE. The insects were placed in pre-weighed centrifuge tubes and then weighed using a Mettler-Toledo electronic balance (EC100 model; 1/10,000 *g* sensitivity). Each treatment and each control was replicated five times with five independent sets of insects.

### qPCR analysis

We isolated total RNA from the five newly-emerged females, using a SV Total Isolation System Kit (model Z3100, Promega Corporation, Madison, WI, USA). First-strand cDNA was synthesized in a 10 µL reaction volume consisting of 0.5 µg of RNA, 0.5 µL of PrimeScriptRT enzyme mix I, 0.5 µL of OligodT primer (50 µM), 2 µL of random hexamers (100 µM), 2 µL 5 × PrimeScriptBuffer (for real time-PCR) and RNase–free dH_2_O up to a final volume of 10 µL, following the PrimeScript RT Kit instructions (TaKaRa Biotechnology, Dalian, China). The cDNA was reverse transcribed using the following program: 37 °C for 15 min, 85 °C for 5 s and 4 °C for 5 min.

We similarly isolated total RNA from the dsRNA-treated and control adults (males or females). Portions (2 μL of the synthesized first-strand cDNA were amplified by qPCR in 20 μL reaction mixtures using a CFX96 real-time PCR system (Bio-Rad Co. Ltd., California, USA). We used two qPCR programs. For *NlPHF7*, 94 °C for 2 min, followed by 40 cycles of 94 °C for 5 s, 60.4 °C for 30 s and 72 °C for 30 s. For *Nlvitellogen* (*Nlvg*), 94 °C for 2 min, followed by 40 cycles of 94 °C for 5 s, 59.7 °C for 30 s and 72 °C for 30 s. *NlPHF7*(NLU003033) and *Nlvg* (AB353856) mRNA levels were separately quantified in relation to the stable expression^14^ of constitutive *actin-1* (EU179846). Primers used for qPCR analysis are listed in Table 2. After amplification, a melting curve analysis was performed in triplicate and the results were averaged. The values were calculated using three independent biological samples and the 2 ^−△△CT^ method^51^ was used for the analysis of relative *NlPHF7* expression level.

### Population growth

A population growth experiment was conducted using the method described by Bao *et al*.^52^ and Ge *et al*.^53^. The experiment set three treatments groups: (1) untreated males (control-♂) × untreated females (control-♀); (2) dsGFP males (dsGFP-♂) × untreated females (control-♀); (3) ds*Nl*PHF7 males (ds*Nl*PHF7-♂) × untreated females (control-♀). The experiment was arranged with a randomized complete block design with five replicates. Two pair of newly emerged *N. lugens* were released to rice plants at the tillering stage covered with a nylon cylindrical cage (20 cm diameter × 80 cm height; screen size: 80-mesh) in each pot. When neonates of the new generation were produced, each treatment group were checked every day and the neonates were counted, then transferred into new plastic plots with the same tillering stage rice plants covered with a nylon cylindrical cage (20 cm diameter × 80 cm height; screen size: 80-mesh) until the original female died. Neonates in the new plastic plot were checked every 2 day until adult emergence, recorded number of females and males, respectively. All adults of next generation were counted (all females plus all males). The rice shoots on which the adults had been feeding were then inspected thoroughly, and the numbers of unhatched eggs were recorded. Hatching rate was also recoded (all adults/all adults plus unhatched eggs). The population growth index (PGI) was expressed by the ratio of N1/N0, which was calculated by dividing the total number (N1) of adults of next generation by the number of adults released (N0 = 4).

### Statistical analysis

Before performing an analysis of variance (ANOVA), data were evaluated for normality and homogeneity of variance using a Bartlett test. Based on these assessments, no transformations were needed. The results presented in figures are expressed as the means ±S.E. Two-way (days post-emergence and dsRNA treatment) ANOVAs were performed to analyze data in Fig. 1. One-way ANOVAs were performed to analyze all other data except for Fig. 1. Multiple comparisons of the means were conducted using Fisher’s Protected Significant Difference (PLSD) test. All analysis were conducted using the data processing system (DPS) of Tang and Feng^54^.
